# Ultrasonographic Assessment with Three-Dimensional Mode of the Urethral Compression Effect following Sling Surgery with and without Mesh Surgery

**DOI:** 10.1155/2019/8285351

**Published:** 2019-01-06

**Authors:** Kun-Ling Lin, Yung-Shun Juan, Shih-hsiang Chou, Cheng-Yu Long

**Affiliations:** ^1^Department of Obstetrics and Gynecology, Kaohsiung Medical University Hospital, Kaohsiung Medical University, Kaohsiung, Taiwan; ^2^Graduate Institute of Clinical Medicine, College of Medicine, Kaohsiung Medical University, No.100, Tzyou 1st Road, Kaohsiung 807, Taiwan; ^3^Department of Urology, Kaohsiung Medical University Hospital, Kaohsiung Medical University, Kaohsiung, Taiwan; ^4^Department of Orthopedics, Kaohsiung Medical University Hospital, Kaohsiung Medical University, Kaohsiung, Taiwan; ^5^Department of Obstetrics and Gynecology, Kaohsiung Municipal Hsiao-Kang Hospital, Kaohsiung Medical University, Kaohsiung, Taiwan

## Abstract

**Background:**

The aim of this study was to assess anatomical changes in the urethra at rest and during straining following sling surgery with and without transvaginal mesh surgery (TVM) in women with stress urinary incontinence (SUI) with or without pelvic organ prolapse (POP) using three-dimensional ultrasonography.

**Methods:**

76 women with SUI with or without pelvic organ prolapse after sling surgery. They underwent sling surgery alone (S group, n=36) or concomitant TVM (M group, n=40). All patients underwent urinalysis, pelvic examinations, urodynamic study, 3D perineal ultrasonography, and personal interviews before and 1 year after surgery. The urethral area was calculated from the axial plane of perineal ultrasonography by multiplying *π* by the long and short axes of the urethral lumen.

**Results:**

The axial area of the middle and distal urethra during straining was significantly smaller than at rest in both groups (P<0.001). In addition, the length of the short axis of the proximal urethra was significantly shorter in those undergoing sling surgery alone during straining compared with those undergoing concomitant sling and mesh surgery (P<0.001).

**Conclusions:**

There was a greater impact on the proximal urethra in women who underwent sling surgery alone than those who underwent sling and TVM surgery together.

## 1. Introduction

Pelvic floor disorders are common in older women, with pelvic organ prolapse (POP) and stress urinary incontinence (SUI) accounting for 80% of pelvic floor dysfunction [1, 2]. The lifetime risk of undergoing surgery for SUI or POP has been reported to be around 11% [3]. With regard to the clinical presentation, these conditions may occur concurrently, or as symptomatic prolapse without SUI or as symptomatic incontinence without obvious prolapse. Bai et al. reported that 63.3% of patients with primary SUI have coexisting POP and that 62.7% of patients with primary POP have coexisting SUI [1].

SUI can be hidden when protruding POP causes the urethra to kink, and occult SUI can be detected from the clinical history and urodynamic reduction test [4]. Accordingly, the rate of postoperative SUI is high at around 51% when a reduction test is positive in preoperative urodynamic studies. Midurethral sling surgery is currently the recommended procedure for SUI [5] and with concomitant sling and transvaginal mesh (TVM) for symptomatic SUI and POP significantly reduced postoperative SUI from previous reports [6–9].

TVM surgery can provide excellent results for women with POP because of a fibrotic effect between the vagina and mesh resulting in good support of the pelvic organs [10]. However, whether there would be less sling compression of the urethra due to the limited mobility of the bladder neck after concomitant TVM is unknown. Clinically, the cure rate of SUI after prolapse surgery concomitant midurethral sling does not seem to be as high as after midurethral sling in women with symptomatic SUI without POP.

The assessment of sling tape using ultrasonography has been widely discussed [11].

However, studies related to interactions between sling and TVM are lacking. Three-dimensional (3D) ultrasonography can provide information on pelvic organ anatomy after sling and/or TVM with simultaneous axial, sagittal, and coronal views [11, 12]. The aim of this study was to compare the urethral compression effect following sling surgery with or without TVM using 3D ultrasonography.

## 2. Patients and Methods

We reviewed the charts of 76 women diagnosed between June 2007 and December 2008 with urodynamic stress incontinence or pelvic organ prolapse with urodynamic stress incontinence or occult SUI, defined as pelvic organ prolapse masking preexisting SUI due to urethral kinking. Occult SUI was diagnosed by clinical history and positive findings on a prolapse reductive test during urodynamic studies.

**Figure 1:**
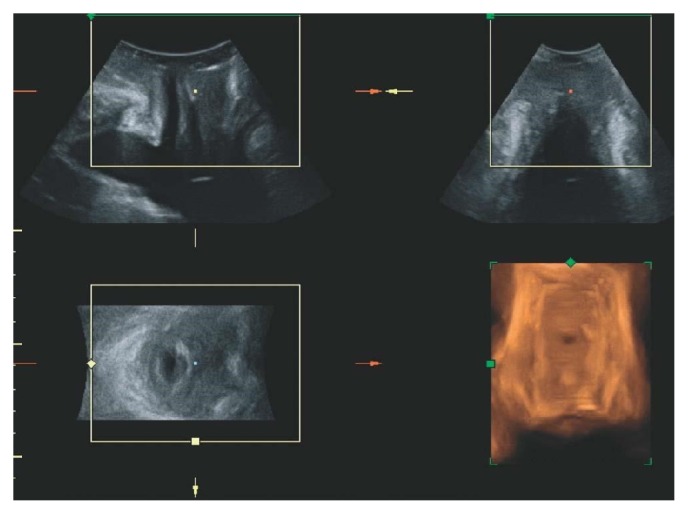
Lower urinary tract on three-dimensional transperineal ultrasound imaging in a woman with tension-free vaginal transobturator tape. The top left image shows the midsagittal view (A-plane), with the tape visible as a hyperechogenic stripe just ventral (left) of the central marker dot. The top right image is a coronal view (B-plane) and the bottom left is the axial or C-plane, showing the tape as a shallow “v” (rotated 90° anticlockwise) to the left of the central marker. The bottom right image is the resulting rendered volume [12].

The patients' history provided clues for occult SUI including (1) incontinence that improved with worsening of pelvic organ prolapse, (2) the need to reposition the prolapse to void, and (3) incontinence with a pessary or reduction of pelvic organ prolapse. Of the 76 women who underwent sling surgery, 40 also underwent transvaginal mesh surgery and 36 did not. Of the 36 women who only underwent sling surgery, 22 underwent tension-free vaginal tape (TVT) (Gynecare TVT System, Ethicon, Inc., Somerville, NJ) surgery and 14 underwent transobturator vaginal tape (TOT) (Monarc; American Medicall Systems, Minnetonka, MN) surgery. The choice of sling type depended on surgeon preference. Of the 40 women who underwent both sling and mesh surgery, 16 underwent TVT surgery and 24 underwent TOT surgery. Routine assessments performed before and 1 year after the procedures included urinalysis, pelvic examinations, and personal interviews using the Bristol Female Lower Urinary Tract Symptoms Questionnaire [13] and 3D perineal ultrasonography (Figure 1).

**Figure 2:**
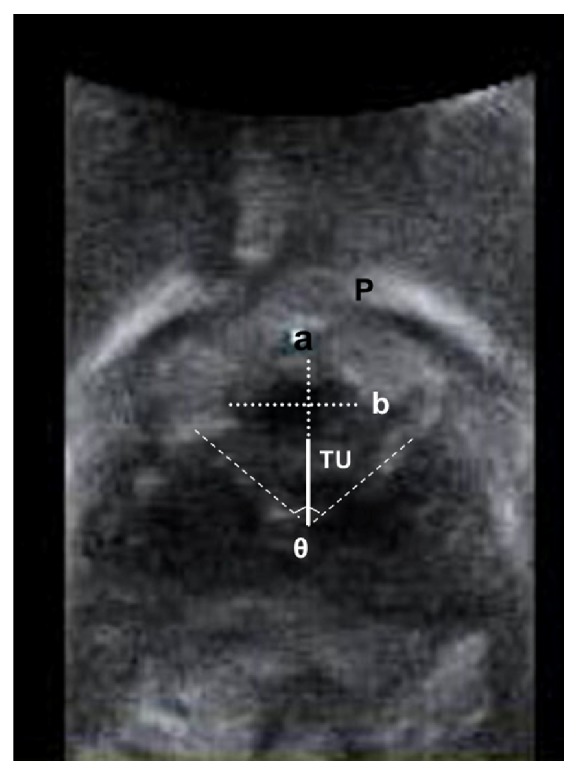
Ultrasound image (axial plane) of the midurethral hypoechoic core in a woman with tension-free vaginal transobturator tape, showing the measurements made for this study: the angle between the two arms of the sling (*θ*), the shortest (a) and longest (b) diameters of the urethral hypoechoic core and the distance between tape and urethra (TU). P, pubis symphysis [12].

We retrospectively analyzed data from a database of mid-urethral sling patients who underwent translabial ultrasound. The Voluson General Electric Sonography, expert 730 type (GE, Healthcare Ultrasound, Zipf, Austria) Ultrasonography was used with 3.5 MHZ curved linear-array transducer placed between the major labia and underneath the external urethral orifice. The assessments included measurement of the angle (*θ*) between the two arms of the sling and calculation of the hypoechoic area of the urethra by multiplying *π* by the lengths of the short and long axes of the urethral core, at rest and during the Valsalva maneuver (Figure 2).

The two investigators (K.L.L. and C.Y.L.) were blinded to each other's results, the type of surgery, and the surgical outcome. They measured every parameter twice and the average of the two measurements was used for statistical analysis. Intraobserver reliability was assessed by the first investigator (K.L.L.), who performed two series of analyses with an interval of 7 to 14 days between them and who was blinded to the previous analysis. Interobserver reliability was assessed by two independent investigators (KLL and CYL). A test-retest series for all parameters showed good interobserver agreement, and the intraclass correlation coefficient ranged from 0.609 to 0.885.

A patient was judged to have been subjectively “cured” when they had no symptoms of SUI on the Bristol Female Lower Urinary Tract Symptoms

Questionnaire and pad test result are less than 2g. The “improved” was defined as the subjective improvement of SUI with pad test > 2g. “Failure” means both no subjective improvement of SUI and pad test>2g. Ethical approval for the retrospective collection of chart data was given by the Institutional Review Board of Kaohsiung Municipal Hsiao-Kang Hospital. All methods were performed in accordance with urogynecologic regulations. Statistical analysis was performed with the paired t-test for continuous variables, and the *χ*2 test and Fisher's exact test for categorical variables. A P value less than 0.05 was considered to indicate statistical significance.

## 3. Results

There were no differences between the sling and sling with mesh groups in body weight, parity, menopausal status, and current use of hormones; however the sling with mesh group was older (Table 1). The older age in the sling with mesh group may have been related to higher parity and menopausal status. Twenty-one patients underwent anterior mesh and 19 underwent both anterior and posterior mesh procedures in the sling with mesh group. Twenty-two patients underwent TVT and fourteen patients underwent TOT in the sling group, compared to sixteen and twenty-four patients, respectively, in the sling with mesh group (Table 2). There were no significant differences in sling type between the two groups. After surgery, the short axis, long axis, and areas of proximal, middle, and distal urethra were similar between the two groups at rest and during the Valsalva maneuver (Table 3). There were also no significant differences in angulation of the two arms of the sling between the two groups at rest and during the Valsalva maneuver. The distance from the tape to the urethra in the two groups at rest and during the Valsalva maneuver was also similar.

The short and long axes and area of the middle urethra significantly decreased from rest to the Valsalva maneuver in both groups (P < 0.001) (Table 4). The short axis of the distal urethra also decreased. However, the short axis of the proximal urethra significantly decreased from rest to the Valsalva compared to a greater extent in the sling group compared to the sling and mesh group (sling: P < 0.001, mesh: P = 0.027). The angulation of the two arms of the sling and the distance from the tape to the urethra significantly differed from rest to the Valsalva maneuver in both groups. One year after surgery, 71 women had improvement of SUI symptoms and were defined as successful treatment, and only five had persistent SUI problems and were defined as having failed treatment (Table 6). The short and long axes and the area of the middle urethra and the short axis of the distal urethra significantly decreased from rest to the Valsalva maneuver in both the successful and failed treatment groups. The significant compression effect from rest to the Valsalva maneuver in the short axis of the proximal urethra in the successful treatment group was found compared to the failed treatment group (P = 0.01/ P = 0.14) (Table 5).

The cure rate of SUI is 80.6% in the sling group and 67.5 % in the mesh group (29/30 versus 27/40, P=0.20). Six patients in the sling group and nine patients in the mesh group are deemed to be improved (6.5% versus 12.4%, P=0.52). One patient in the sling group and four patients in the mesh group defined failure (14% versus 5.2 %, P=0.36). The efficacy of surgery is 97.2% (35/36) in the sling group and 90 % (36/40) in the mesh group (Table 6).

## 4. Discussion

Long et al. [14] and Yang et al. [15] reported that TVT seems to undergo greater urethral compression than TOT or TVTO in ultrasound findings [16]. Differences in the sling type may therefore result in different cure rates of SUI [17]. In the current study, the percentage of patients receiving TVT and TOT was similar in both groups, so variability in sling type had a minimal effect on the cure rate of SUI.

Long et al. [18] reported that the bladder neck was localized more cranially with the use of a tension-free bladder neck sling when straining is compared to the preoperative condition. It is known that transvaginal synthetic mesh reconstruction provides cystocele support from the bladder to the bladder base due to a mesh fibrosis and stiffness effect, and this seems to limit the mobility of the bladder when straining. Hammock hypothesized [19] that abdominal pressure causes the urethra and bladder to descend against the supportive tissue of the vagina, thereby compressing the urethra causing it to close to prevent urinary leakage. Therefore, poor bladder mobility after transvaginal mesh surgery may explain the lesser compressive effect of the sling during the Valsalva maneuver. Lo et al. [20] reported that the mobility of the mesh and/or bladder neck had an important role in determining the success rate of middle urethral sling surgery in sonography findings. In the current study, the lesser compressive effect was reflected in the short axis of the proximal urethra in the sling and mesh group.

Consistent with the findings of Huang et al. [21], the success rate of treating SUI was comparable in the sling and sling and transvaginal mesh groups, although in ultrasound findings there was a higher incidence of proximal urethral compression from rest to straining in the women who only underwent sling surgery. We also found that a lesser compressive effect was detected in the short axis of the proximal urethra in the failed treatment group compared to the successful treatment group. A lesser sling compressive effect on the urethra in the postmesh surgery group may have been reflected in a lower cure rate of SUI compared to the sling only group; however this was not detected. It is possible that a higher number of cases or other urethral compression parameters can reflect subtle differences in sling compression in both the sling and sling and mesh groups.

Huang et al. [21] reported that the short-term success rates with slings in women who underwent TOT with or without anterior vaginal mesh were similar. In contrast, Stav et al. [22] reported that concurrent prolapse surgery was a major risk factor for recurrent SUI after 1 year. In the current study, greater compression of the short axis in the proximal urethra was only found in the sling surgery group, and more obvious significant differences between the two groups may have been found with more patients or a longer study period [6].

Yang [23] reported that the effectiveness of the sling depends on the mechanical interaction between the tape and urethra and that failure of a sling is related to a lack of urethral encroachment (kinking) at rest, bladder neck funneling, a urethral location of less than the 50th percentile, and a resting tape angle of less than 165 degrees, and they reported a high kinking rate in the successful treatment group compared to the failed treatment group (62% versus 15%). In the current study, we focused on the hypoechogenic area of the urethra and the distance between the tape and urethra at rest and during straining. It would also have been interesting to include more sonography parameters to highlight the differences between sling surgery with and without the use of anterior wall mesh.

The limitation of this study is that the retrospective nature may also have selection bias even though women who presented for treatment were included with usage of objective data. Otherwise the treatment choice of stress urinary incontinence depends on the surgeon's preference. The compression effect of midurethral slings may be different though the report of Ross S [24], the subjectively successful rate of retropubic, and transobturator slings are similar. Furthermore, women with the intrinsic sphincter deficiency of SUI should be identified to lessen the bias of severity of SUI.

## Figures and Tables

**Table 1:** Clinical background in both groups.

	S (n=36)	S+M (n=40)	P value
Age (years)	54.4±8.1	61.7±9.4	<0.01^a^
Body weight (kg)	62.4±8.7	58.2±10.2	0.06^a^
Parity	2.8±1.2	3.3±1.3	0.07^a^
Menopause	24(66.7)	36(90.0)	0.022^b^
HT	4 (11.1)	3 (7.5)	0.70^b^
Previous hysterectomy	7 (19.4)	6 (15.0)	0.61^c^
Concomitant procedures			
Anterior mesh		21(52.5)	
Anterior and posterior mesh		19(47.5)	

Values are given as mean ± standard deviation or n(%); HT= hormone therapy.

ISD = intrinsic sphincter deficiency.

a. Unpaired t-test, b. Fisher's exact test, and c. Chi-square test.

**Table 2:** Clinical background in both groups.

	S (n=36)	S+M (n=40)	P value

TVT	22(61.1)	16(40.0)	0.07
TOT	14(38.9)	24(60.0)	

Values are given as mean ± standard deviation or n(%)*χ*2 test.

**Table 3:** Postoperative urethral topography at rest and during straining in both groups.

	Resting			Straining		
	S (n=36)	S+M (n=40)	P-value*∗*	S (n=36)	S+M (n=40)	P-value*∗*
Proximal (area)(mm^2^)	140.9±57.4	135.6±47.7	0.66	122.0±60.6	122.2±55.5	0.99
a (mm)	6.2±1.6	5.8±1.2	0.21	5.4±1.5	5.4±1.2	0.87
b (mm)	7.0±1.6	7.4±2.0	0.38	6.8±1.8	7.0±2.2	0.64
Middle (area)(mm^2^)	134.0±72.7	147.9±69.3	0.40	88.5±45.4	90.7±48.4	0.84
a (mm)	5.5±1.8	5.9±2.0	0.42	4.4±1.4	4.5±1.4	0.78
b (mm)	7.3±2.5	7.8±2.1	0.27	6.1±2.1	6.1±1.7	0.97
Distal (area)(mm^2^)	112.3±63.6	130.9±82.2	0.27	80.7±50.3	93.4±52.9	0.28
a (mm)	5.0±1.7	5.4±2.1	0.38	4.2±1.2	4.5±1.3	0.21
b (mm)	6.7±2.3	7.3±2.9	0.30	5.9±2.8	6.3±2.3	0.51
2-arm angle (°)	102.9±17.4	110.0±15.9	0.04	89.4±19.2	93.9±19.3	0.32
TU	5.3±1.8	5.9±2.4	0.20	4.3±1.7	4.9±2.2	0.14

S, sling; S+M, sling plus mesh; °, degree; TU, distance between the tape and urethra. *∗*Statistical significance; Student's t-test; #*χ*2 test.

**Table 4:** Postoperative ultrasound measurements from resting to straining in both groups.

	S (n=36)			S+M (n=40)		
	Resting	straining	P-value	Resting	straining	P-value
Proximal (area)(mm2)	140.9±57.4	122.0±60.6	0.009*∗*	135.6±47.7	122.2±55.5	0.05*∗*
a (mm)	6.2±1.6	5.4±1.5	<0.001*∗*	5.8±1.2	5.4±1.2	0.027*∗*
b (mm)	7.0±1.6	6.8±1.8	0.34	7.4±2.0	7.0±2.2	0.19
Middle (area )(mm2 )	134.0±72.7	88.5±45.4	<0.001*∗*	147.9±69.3	90.7±48.4	<0.001*∗*
a (mm)	5.5±1.8	4.4±1.4	<0.001*∗*	5.9±2.0	4.5±1.4	<0.001*∗*
b (mm)	7.3±2.5	6.1±2.1	<0.001*∗*	7.8±2.1	6.1±1.7	<0.001*∗*
Distal (area )(mm2)	112.3±63.6	80.7±50.3	<0.001*∗*	130.9±82.2	93.4±52.9	<0.001*∗*
a (mm)	5.0±1.7	4.2±1.2	0.002*∗*	5.4±2.1	4.5±1.3	0.001*∗*
b (mm)	6.7±2.3	5.9±2.8	0.002*∗*	7.3±2.9	6.3±2.3	0.02*∗*
2-arm angle (°)	102.9±17.4	89.4±19.2	<0.001*∗*	110.0±15.9	93.9±19.3	<0.001*∗*
TU	5.3±1.8	4.3±1.7	<0.001*∗*	5.9±2.4	4.9±2.2	<0.001*∗*

S, sling; S+M, sling plus mesh; °, degree; TU, distance between the tape and urethra. *∗*Statistical significance; paired t-test

**Table 5:** Postoperative ultrasound measurements in women reporting success and failure outcome.

	Success (n=71)			Failure (n=5)		
	Resting	Straining	P-value	Resting	Straining	P-value
Proximal (area)(mm2 )	47.5±15.4	44.1±15.8	0.11	43.3±11.7	41.3±20.9	0.76
a (mm)	6.3±1.4	5.7±1.1	0.01*∗*	6.0±1.4	5.3±1.0	0.14
b (mm)	7.5±1.7	7.5±1.6	0.98	7.3±0.9	7.7±3.0	0.73
Middle (area )(mm2 )	49.6±19.1	32.3±11.7	<0.01*∗*	59.9±14.0	29.2±7.0	<0.01*∗*
a (mm)	6.2±2.0	4.8±1.1	<0.01*∗*	6.6±0.9	4.6±0.5	<0.01*∗*
b (mm)	8.0±1.9	6.8±1.7	<0.01*∗*	9.0±1.3	6.3±1.3	<0.01*∗*
Distal (area )(mm2 )	45.6±24.3	34.3±17.1	<0.01*∗*	52.0±25.5	29.2±7.4	0.04*∗*
a (mm)	5.7±2.1	4.6±0.9	<0.01*∗*	5.4±0.8	4.6±1.2	0.055
b (mm)	7.8±2.6	7.2±2.7	0.18	9.2±3.1	6.5±1.4	0.07
2-arm angle (°)	106.7±15.7	89.1±15.9	<0.01*∗*	112.6±9.4	94.5±14.8	<0.01*∗*
TU	5.7±2.1	4.5±1.8	<0.01*∗*	4.8±2.4	3.8±2.1	<0.01*∗*

TVT, tension-free vaginal tape; TVT-O, TVT-obturator tape; °, degree; TU, distance between the tape and urethra. *∗*Statistical significance; paired t-test

**Table 6:** Operative results and complications.

	S (n=36)	S+M (n=40)	P value
Cure rates	29(80.6)	27(67.5)	0.20a
Improved	6(6.5)	9(12.4)	0.52a
Failure	1(14.0)	4(5.2)	0.36b
Efficacy of Surgery	35(97.2)	36(90.0)	0.36b

Values are mean ± standard deviation or n (%).

a. *χ*2 test.

b. Fisher's exact test.

## Data Availability

The data used to support the findings of this study are available from the corresponding author upon request.
